# Comparing the Efficacy of Intra-articular Platelet-Rich Plasma and Corticosteroid Injections in the Management of Frozen Shoulder: A Randomized Controlled Trial

**DOI:** 10.7759/cureus.39728

**Published:** 2023-05-30

**Authors:** Tarun Kumar Somisetty, Hariprasad Seenappa, Subhashish Das, Arun H Shanthappa

**Affiliations:** 1 Orthopedics, Sri Devaraj Urs Medical College, Kolar, IND; 2 Pathology, Sri Devaraj Urs Medical College, Kolar, IND

**Keywords:** quickdash, spadi, intraarticular corticosteroid injection, intraarticular platelet-rich plasma injection, frozen shoulder, periarthritis shoulder

## Abstract

Introduction

Periarthritis of the shoulder, or frozen shoulder (FS), is a common, painful, and disabling condition with varied treatment strategies. Intra-articular (IA) corticosteroid (CS) injections are a popular treatment option, but their efficacy is often temporary. Platelet-rich plasma (PRP) has emerged as an alternative therapy for adhesive capsulitis, but the literature on its effectiveness is limited. This study aimed to compare the efficacy of IA PRP and CS injections in managing FS.

Methods

In this prospective, randomized study, 68 patients who met the inclusion criteria were enrolled and randomized using a computer-generated table into two groups: Group 1 (IA PRP) received 4 ml PRP, and Group 2 (IA CS) received 2 ml (80 mg) of methylprednisolone acetate mixed with 2 ml normal saline (for a total of 4 ml) as a CS injection in the IA area of the shoulder. Outcome measures included pain; shoulder range of motion (ROM); the condensed version of the disabling conditions of the arm, shoulder, and hand (QuickDASH) score; and the shoulder pain and disability index (SPADI) score. Participants were monitored via follow-up for 24 weeks, with pain and function assessed at each evaluation using the visual analog scale (VAS) score, the SPADI score, and the QuickDASH score.

Results

The IA PRP injections demonstrated better long-term outcomes than the IA CS injections, significantly improving pain, shoulder ROM, and daily activity performance. After 24 weeks, the mean VAS score in the PRP and methylprednisolone acetate groups was 1.00 (1.0 to 1.0) and 2.00 (2.0 to 2.0), respectively (P≤0.001). The mean QuickDASH score was 41.83 ± 6.33 in the PRP group and 48.76 ± 5.08 in the methylprednisolone acetate group (P≤0.001). The mean SPADI score was 53.32 ± 7.49 in the PRP group and 59.24 ± 5.80 in the methylprednisolone acetate group (P≤0.001), indicating a significant improvement in the PRP group's pain and disability scores after 24 weeks. The rate of complications was similar between the two groups.

Conclusions

Our findings suggest that IA PRP injections provide better long-term results than IA CS injections for managing FS. Platelet-rich plasma can be used as a treatment modality for better outcomes, particularly when the patient is contraindicated or refuses CS treatment. Further research is needed to evaluate the efficacy of these treatment modalities at different stages of FS and explore the potential benefits of ultrasound-guided injections.

## Introduction

Periarthritis of the shoulder, also known as frozen shoulder (FS) or adhesive capsulitis, is characterized by chronic shoulder pain and restricted range of motion (ROM) [1]. There are two subtypes of FS: primary (idiopathic) and secondary. A secondary FS results from an accident, rotator cuff dysfunction, impingement, cardiovascular illness, hemiparesis, or diabetes [2]. The annual incidence of FS in the general population ranges from 3% to 5%, and in individuals with diabetes, it can reach 20% [3].

In modifying a patient's perception of their general health, the burden of shoulder pathologies has been ranked as high as the burden of having congestive heart failure, hypertension, acute myocardial infarction, diabetes mellitus, and/or depression [4]. Previously considered self-limiting, recent literature reveals that the course of the disease might last as long as 10 years, with up to 40% of patients continuing to suffer from it throughout their lives [5].

Treating a frozen shoulder often involves temporary relief through intra-articular (IA) corticosteroid (CS) injections. A randomized control study has shown that IA CS results in pain relief and improved ROM in patients suffering from FS [6]. However, recent advancements have introduced platelet-rich plasma (PRP) as a promising alternative therapy. Platelet-rich plasma is an autologous blood fraction that is easy to use, quickly prepared, and minimally invasive. It comprises concentrated platelets and growth factors that deliver growth-promoting cytokines and other molecular derivatives to the injury site, promoting healing [7]. In recent studies, IA PRP injections were used as a treatment modality for managing FS, showing improved pain and ROM during long-term follow-up [8].

Therefore, the current randomized controlled study aims to determine the clinical efficacy of IA PRP and IA CS in FS in terms of pain relief and improvement of ROM using the visual analog scale (VAS) score; the shoulder pain and disability index (SPADI) score; and the condensed version of the disabling conditions of the arm, shoulder, and hand (QuickDASH) score.

## Materials and methods

A prospective, randomized, controlled hospital-based observational follow-up study was conducted at the R L Jalappa Hospital and Research Center (Kolar, India), orthopedics department from December 2020 to July 2022. The study was approved by the institutional ethics committee of Sri Devaraj Urs Medical College, Kolar, India (approval no. DMC/KLR/IEC/740/2022-23), and written informed consent was obtained from all participants. Participants were randomly divided into two groups using a computer-generated randomization table. Group 1 (IA PRP) received 4 ml PRP, while Group 2 (IA CS) received 2 ml (80 mg) of methylprednisolone acetate mixed with 2 ml normal saline (for a total of 4 ml) as a CS injection into the shoulder's IA area.

Patients of either sex aged 18 to 75 years with a clinical diagnosis of FS who desired a higher level of activity but could not achieve it even after three months of conservative treatment were included in the study. Patients on antiplatelet, anticoagulant therapy, antitumor or immunosuppressive drugs, uncontrolled diabetes, shoulder instability, immunodeficiency, local skin lesions, or a history of trauma or surgery were excluded from the study. Participants in both groups were assessed at the end of two, four, eight, 12, and 24 weeks following the procedure.

Statistical analysis

Data were entered into a Microsoft Excel (Microsoft Corp., Redmond, WA, USA) sheet, and statistical analysis was performed using SPSS Statistics version 24.0 (IBM Corp., Armonk, NY, USA). The descriptive analysis presented the relevant statistics (mean ± SD) for quantitative variables, while frequency and percentage were used for categorical variables. The independent sample t-test was used to determine the mean values of quantitative parameters that were normally distributed among the two study groups. For non-normally distributed quantitative parameters, medians and interquartile ranges were compared between the two study groups using the Mann-Whitney U test. We considered P<0.05 as statistically significant.

## Results

The mean age was 58.3 ± 8.1 years in the IA PRP group and 58.5 ± 7.7 years in the IA CS group, with no statistically significant difference between the two study groups (P=0.9394). In the IA PRP group, 15 participants (44.12%) were male, and 19 (55.88%) were female, while in the IACS group, 20 participants (58.82%) were male, and 14 (41.18%) were female. The difference in sex proportion between the study groups was not statistically significant (P=0.2251). In the IA PRP group, 18 (52.94%) reported right-side involvement, and 16 (47.06%) reported left-side involvement. In the IA CS group, 23 (67.65%) reported right-side involvement, and 11 (32.35%) reported left-side involvement (P=0.2153). There was no statistically significant difference in the proportion of duration (months) between the study groups (P=0.105). In the IA PRP group, 12 participants (35.29%) had diabetes, while 14 (41.18%) had diabetes in the IA CS group. The difference between the two study groups was not statistically significant (P=0.6177). Eight participants (23.53%) had hypertension in the IA PRP group, while two (5.88%) had hypertension in the IA CS group. The difference between the two study groups was not statistically significant (P=0.0832). One participant (2.94%) in the IA CS group, and two participants (5.88%) in the IA PRP group experienced complications, but the difference was not statistically significant (P=1.00, Table 1).

**Table 1:** Study population demographic data (n=68), with 34 in each group IA: Intra-articular, PRP: Platelet-rich plasma, CS: Corticosteroid

Parameter	Type	Group 1 (IA PRP), n (%)	Group 2 (IA CS), n (%)	P-value
Sex	Male	15 (44.12%)	20 (58.82%)	0.2251
	Female	19 (55.88%)	14 (41.18%)	
Side	Right	18 (52.94%)	23 (67.65%)	0.2153
	Left	16 (47.06%)	11 (32.35%)	
Duration	<6 Months	12 (35.29%)	7 (20.59%)	0.105
	6-12 Months	10 (29.41%)	14 (41.18%)	
	12-18 Months	10 (29.41%)	5 (14.71%)	
	18-24 Months	2 (5.88%)	6 (17.65%)	
	>24 Months	0 (0%)	2 (5.88%)	
Comorbidities	Diabetes	12 (35.29%)	14 (41.18%)	0.6177
	No Diabetes	22 (64.71%)	20 (58.82%)	
	Hypertension	8 (23.53%)	2 (5.88%)	0.0832
	No Hypertension	26 (76.47%)	32 (94.12%)	
Complications	Present	2 (5.88%)	1 (2.94%)	1.000
	Absent	32 (94.12%)	33 (97.06%)	

The median difference in VAS between the study groups at pre-injection and eight weeks was not statistically significant (P>0.05). However, a significant difference was found in VAS at post-injection, two, four, 12, and 24 weeks (Table 2). 

**Table 2:** VAS scores between the groups over time (n=68) IA: Intra-articular, PRP: Platelet-rich plasma, CS: Corticosteroid, IQR: Interquartile range, VAS: Visual analog scale

Parameters	Group 1 (IA PRP), n=34, median (IQR)	Group 2 (IA CS), n=34, median (IQR)	Mann-Whitney U-test P-value
Pre-injection (VAS score)	8.50 (8.0 to 9.0)	8.00 (7.0 to 9.0)	0.1439
Post-injection (VAS score)	7.00 (7.0 to 8.0)	6.00 (5.25 to 7.0)	<.001
VAS at 2 weeks	6.00 (6.0 to 7.0)	5.00 (4.25 to 6.0)	<.001
VAS at 4 weeks	5.00 (5.0 to 6.0)	5.00 (4.0 to 5.0)	0.0021
VAS at 8 weeks	4.00 (3.0 to 4.0)	4.00 (3.0 to 4.0)	0.4766
VAS at 12 weeks	2.00 (2.0 to 3.0)	3.00 (2.0 to 3.0)	0.0011
VAS at 24 weeks	1.00 (1.0 to 1.0)	2.00 (2.0 to 2.0)	<.001

**Figure 1:**
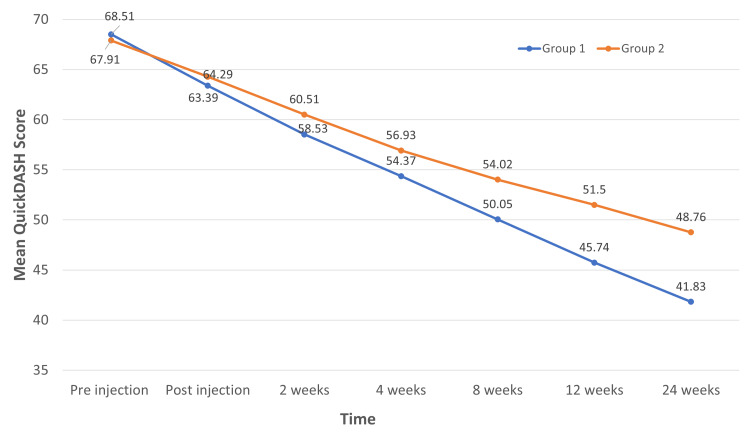
: Line chart of QuickDASH score between groups over time (n=68) Both study groups experienced a progressive decline in QuickDASH scores, while Group 1 (IA PRP) experienced a considerable decline at eight, 12, and 24 weeks. QuickDASH: Condensed version of the disabling conditions of the arm, shoulder, and hand score; IA: Intra-articular; PRP: Platelet-rich plasma

The mean difference between the study groups in QuickDASH at pre-injection, post-injection, two weeks, and four weeks was not statistically significant (P>0.05). However, a significant difference was found in QuickDASH at eight weeks, 12 weeks, and 24 weeks (Figure 1). 

**Figure 2:**
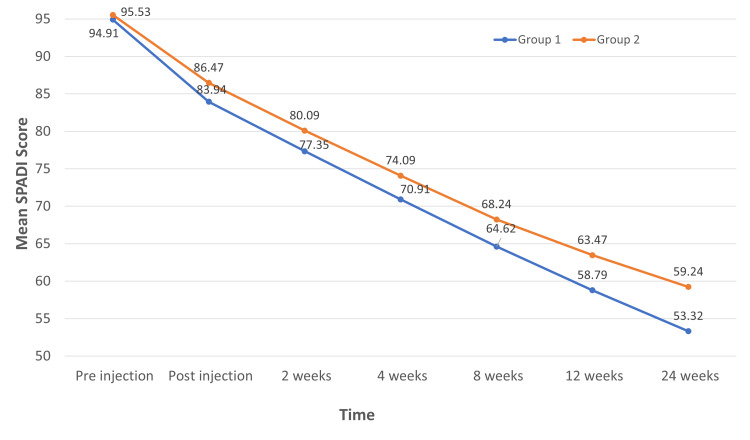
Line chart of SPADI score between groups over time (N=68) The SPADI score declined in both groups. But after eight, 12, and 24 weeks, Group 1 (IA PRP) had a significantly lower SPADI score than Group 2 (IA CS). SPADI: Shoulder pain and disability index, IA: Intra-articular, PRP: Platelet-rich plasma, CS: Corticosteroid

The mean difference in SPADI between the study groups at pre-injection, post-injection, two weeks, and four weeks was not statistically significant (P>0.05). However, a significant difference was discovered in SPADI at eight weeks, 12 weeks, and 24 weeks (Figure 2).

## Discussion

Frozen shoulder is a common, painful, and incapacitating ailment that is self-limiting. It has three clinical phases: (1) the painful freezing phase, lasting 10 to 30 weeks; (2) the adhesive/frozen phase, occurring four to 12 months after disease onset; and (3) the resolution/thawing phase, developing 12 to 42 months after disease onset. Flexion and abduction movements are more restricted than other shoulder joint movements [9]. Approximately 70% of total FS patients are female, and the dominant arm is more commonly affected than the non-dominant arm [10]. Those who have previously experienced FS have a 5% to 34% probability of developing it in the opposite shoulder at some point. Synchronous bilateral involvement occurs in approximately 14% of patients [11].

Up to 90% of patients with FS benefit greatly from conservative treatment, with the method often used depending on the clinicopathological stage of the condition. Common conservative management options include oral medications like non-steroidal anti-inflammatory drugs (NSAIDs), calcitonin, CS, physical therapy, exercise, IA steroid injections, and hydrodilation. Only a few patients who do not improve with conservative therapy require surgical intervention, such as manipulation under anesthesia or arthroscopic capsular release [12].

The etiology, treatment strategy, and treatment choice for FS remain debated. Although steroid injections are the most popular form of therapy, they provide only temporary pain relief and ROM improvement and are associated with complications [13]. Recently, PRP has gained popularity as an alternative to steroids for treating FS, particularly when patients have rejected or are contraindicated for steroid use. Preliminary evidence suggests that PRP injections are associated with better functional outcomes [14].

According to Lin et al., PRP injection was more effective and longer-lasting than IA CS injection [15]. The researchers attributed the anti-inflammatory and analgesic effects as the factors responsible. Platelet-rich plasma also promotes soft tissue revascularization and increases the local concentration of growth factors to enhance and accelerate recovery [15].

In our study population, FS affected males more frequently than females (ratio 1.06:1). This contrasts with the findings of Upadhyay et al. [14], who reported female predominance and involvement of the non-dominant side compared to the dominant side. Kothari et al. [3] had a similar age range in their study population (mean age 51.9 ± 10.1 years) but found female dominance, and the majority had dominant side involvement (58.9%).

Most of our study population experienced symptoms for six to 12 months, followed by less than six months and 12 to 18 months, indicating that most patients presented in the freezing and frozen stages. The proportion of comorbidities, such as diabetes and hypertension, was insignificant between the groups. Diabetes mellitus (38%) was the most common comorbidity in the study population, followed by hypertension (15%), which is consistent with the findings of Gupta et al. [6].

Our study results align with Buchbinder et al. [16] and Wang et al. [17], who found that steroid injections for FS could be helpful, even if their effects might be transient and not long-lasting. Upadhyay et al. [14] found that PRP injections had a long-term effect on pain relief and improved shoulder function, lasting about 12 weeks.

Aslani et al. [18] also reported similar findings to our study, demonstrating improvements in QuickDASH score, reduction in shoulder pain, and improvement in ROM when assessing the role of PRP in managing FS. Ünlü et al. [19] evaluated the efficacy of IA PRP injection versus normal saline in managing FS and found improvements in SPADI score and reductions in VAS score, similar to our results obtained in the PRP group.

Platelet-rich plasma has chemotactic and mitogenic properties and functions as a growth factor agonist. The combination of large amounts of activated platelets, growth factors, and anti-inflammatory compounds alters the healing cascade and inflammatory pathway, reversing the degenerative process [6,14,20]. Our study found a significant decrease in SPADI scores at all follow-up intervals.

Yoon et al. demonstrated no appreciable changes in the effectiveness of CS at various levels, suggesting a preference for initially using a low dose and tapering off its effects in long-term follow-up [21]. To determine whether PRP can be used for long-term follow-up in managing FS, a major limitation for IA CS, a study assessing PRP in FS found relief of symptoms even after 12 weeks [22]. The rate of complications observed in our study was similar to those reported by Kothari et al. [3], Barman et al. [20], and Upadhyay et al. [14].

Despite the meticulously designed protocol of our study, there are some limitations. All stages of FS were included in the study, so further research is needed to evaluate the efficacy of these treatment modalities in different stages of FS. This prospective randomized study is purely subjective (SPADI score) as no attempts were made to examine the repair using imaging methods like MRI or histopathology assessment. Future research may investigate the role of ultrasound-guided injections in the musculoskeletal system.

## Conclusions

Our study demonstrated that IA PRP injection resulted in better outcomes i.e., decreased pain, improved shoulder ROM, and increased ability to perform daily activities for patients with FS compared to IA CS injection. Platelet-rich plasma showed better long-term outcomes than CS, suggesting that PRP can be used as a treatment modality in managing FS for better results. We emphasize the emerging importance of PRP in treating chronic musculoskeletal disorders like FS, particularly in situations where the patient refuses or is contraindicated for CS treatment.
